# Oxaliplatin-induced neuropathy: the preventive effect of a new super-oxide dismutase modulator

**DOI:** 10.18632/oncotarget.27248

**Published:** 2019-11-05

**Authors:** Marie-Anne Guillaumot, Olivier Cerles, Hélène C. Bertrand, Evelyne Benoit, Carole Nicco, Sandrine Chouzenoux, Alain Schmitt, Frédéric Batteux, Clotilde Policar, Romain Coriat

**Affiliations:** ^1^ Département “Development, Reproduction and Cancer”, Institut Cochin, Paris Descartes Université, Sorbonne Paris Cité, INSERM U1016, Paris, France; ^2^ Laboratoire des Biomolécules, LBM, Département de Chimie, École Normale Supérieure, PSL University, Sorbonne Université, CNRS, Paris, France; ^3^ Service d’Ingénierie Moléculaire des Protéines (SIMOPRO), CEA de Saclay, Université Paris-Saclay, Gif-sur-Yvette, France; ^4^ Institut des Neurosciences Paris-Saclay (Neuro-PSI), CNRS, UMR CNRS/Université Paris-Sud 9197, Université Paris-Saclay, Gif-sur-Yvette, France; ^5^ Plateforme Imagerie Cellulaire, Microscopie électronique Institut Cochin, Université Paris Descartes, Sorbonne Paris Cité, INSERM U1016, Paris, France; ^6^ Service d’Immunologie, Centre Hospitalo-Universitaire Cochin AP-HP, Université Paris Descartes, Paris, France; ^7^ Service de Gastro-Entérologie du Centre Hospitalo-Universitaire Cochin, APHP, Université Paris Descartes, Paris, France

**Keywords:** oxidative stress, super oxide dismutase, oxaliplatine, cancer cell, chemotherapy toxicities

## Abstract

By using the differential in level of oxidative status between normal and cancer cells, SuperOxide Dismutase (SOD) mimetics can have anti-tumor efficacy and prevent oxaliplatin-induced peripheral neuropathy. Our objective was to evaluate the neuroprotective efficacy of MAG, a new SOD mimic.

*In vitro*, the effects of MAG alone or with oxaliplatin were studied on colon cancer cells (HT29 and CT26) and on normal fibroblast cells (NIH3T3). The cell viability (by crystal violet) as well as the production of reactive forms of oxygen and glutathione (by spectrofluorimetric assay) was measured. *In vivo*, efficacy on tumor growth was assessed in mice grafted with CT26 colon cancer cells. The effects on induced neurotoxicity were measured by specific behavioral Von Frey nociception, cold-plate tests, specific functional neuromuscular assay and electron microscopy.

*In vitro*, MAG induced a production of hydrogen peroxide in all cells. At 24 h-incubation, MAG exhibits a cytotoxic activity in all cell lines. A cytotoxic additive effect of MAG and oxaliplatin was observed through oxidative burst. *In vivo*, oxaliplatin-treated mice associated with MAG did not counteract oxaliplatin’s antitumoral efficacy. After 4 weeks of treatment with oxaliplatin combined with MAG, behavioral and functional tests showed a decrease in peripheral neuropathy induced by oxaliplatin *in vivo*. Electron microscopy analyses on sciatic nerves revealed an oxaliplatin-induced demyelination which is prevented by the association of MAG to this chemotherapy.

In conclusion, MAG prevents the appearance of sensitive axonal neuropathy and neuromuscular disorders induced by oxaliplatin without affecting its antitumor activity.

## INTRODUCTION

Low levels of Reactive Oxygen Species (ROS) regulate cellular signaling and play an important role in normal cell proliferation by activating growth-related signaling pathways [1, 2]. In cancer cells, higher levels of ROS with inhibition of anti-oxidant systems compared to normal cells have been observed [3]. In tumoral cells, DNA damage induced by increased ROS production leads to genomic instability and cancer progression [4]. By contrast, high levels of ROS appear to activate and modulate apoptosis when cells are under stress conditions by promoting pro-apoptotic signaling molecules including P53-induced apoptosis [5, 6]. Anti-cancer agents, such as oxaliplatin [7], have been shown to produce a burst in intracellular oxidative stress, that leads to cancer cells’ death through a higher sensitivity to the increased oxidative stress.

Peripheral neuropathy is oxaliplatin’s main limiting factor, even in cases where its anti-tumoral efficacy remains [8]. The pathogenic events leading to the oxidative burst have been proven to be involved in the pathophysiology of the neurotoxicity induced by oxaliplatin [9].

Several compounds with properties of redox balance modulation have been developed with promising results in counteracting oxaliplatin-induced neurotoxicity [10–13]. Indeed, compounds able to mimic Manganese SuperOxide Dismutase (MnSOD), an endogenous mitochondrial anti-oxidant enzyme with a prominent role as an anti-oxidant, are promising due to their ability to catalyze the dismutation of superoxide (O_2_^−^) without additional oxidative stress if manganese is released, compared to other ions [14–17]. Some of them have proven their efficacy in oxaliplatin-induced neurotoxicity in un-blinded, non-randomized clinical studies [11, 18]. In Coriat *et al* [11], 22 cancer patients with grade ≥ 2 oxaliplatin-associated CIPN received intravenous Mangafodipir following oxaliplatin. In 77% of the patients additionally treated with mangafodipir, oxaliplatin-induced neurotoxicity improved or stabilized after four cycles. After eight cycles, oxaliplatin-induced neurotoxicity was downgraded in six of seven patients. In the second clinical phase II trial with calmangafodipir [18], Calmangafodipir-treated patients had a less severe neurotoxicity, significantly less cold allodynia and significantly fewer sensory symptoms. While encouraging, there is no randomized clinical trials confirming the efficacy observed in the phase 2 studies [19].

**Figure 1:**
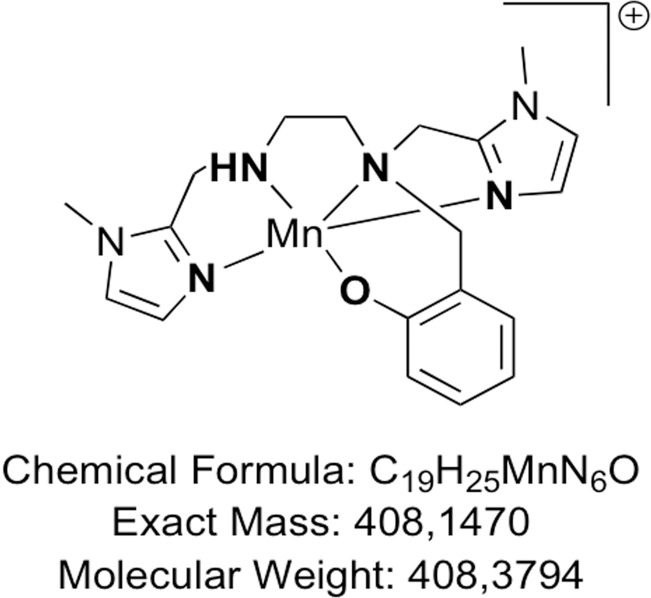
The chemical structure of the 1,2-diaminoethane-based Mn (II) complex.

The use of proteins for therapeutics can be limited by several factors, including low cellular penetration, short half-life, and immunogenicity [20]. These conditions can be overcome by using low molecular-weight redox-active complexes mimicking SOD activity [14, 21–23]. Complex MAG, a low-molecular-weight MnSOD mimic molecule (Figure 1) bioinspired from the active site of MnSOD [23, 24] was shown to be an effective stable compound with excellent intracellular diffusion compared to other larger SOD-mimic Mn-complexes, such as Mangafodipir [25]. Furthermore, MAG was recently shown to have an anti-inflammatory activity in epithelial cells (HT29) genetically modified to generate an important inflammation upon bacterial lipopolysaccharide (LPS) activation [26].

As these anti-oxidant properties were promising, we have chosen to explore its activity in a cancer model to evaluate the potential antitumoral effect of a 1,2-diaminoethane-based Mn (II) complex, and its neuroprotective effect when associated with oxaliplatin. We hypothesized that MAG could prevent sensitive oxaliplatin-induced neuropathy when associated with oxaliplatin by increasing the dismutation of superoxide in hydrogen peroxide and by maintaining higher GSH levels in normal cells. Our aim was to evaluate the effect of MAG on the modulation of the redox balance, its cytotoxicity with or without oxaliplatin, and to investigate the antitumoral and neuroprotective effects of MAG *in vivo* in a mice model.

## RESULTS

### MAG increases H_2_O_2_ production and decreases GSH at 24 h in all cell lines through O_2_− dismutation by SOD mimic activity

**Figure 2:**
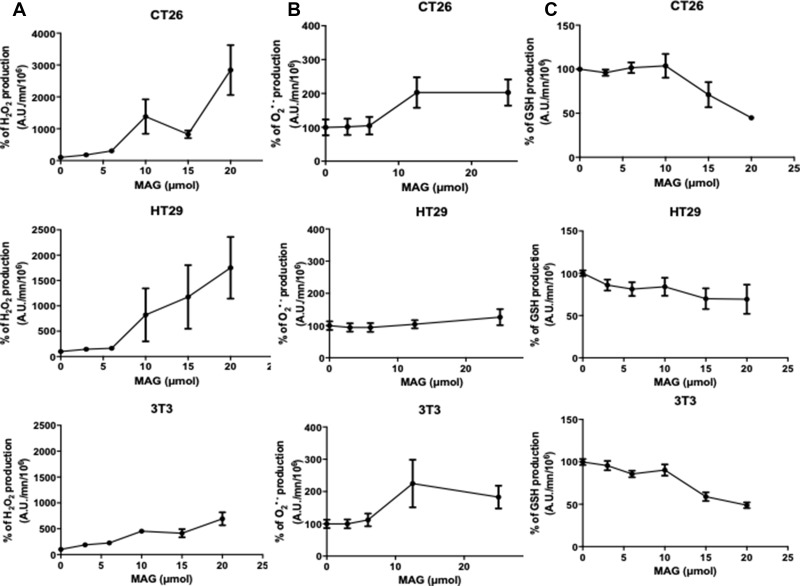
The *in vitro* ROS production after 24 h a 6 h incubation of MAG in CT26, HT29 and NIH3T3 cells. (**A**) Hydrogen peroxide production was assessed during 6 h after incubation of CT26, HT29 and NIH3T3 cells during 24 h with 2’,7’-dichlorodihydrofluorescein diacetate with increasing amounts of MAG. (**B**) Hydroxyl radical production was assessed during 6 h after incubation of CT26, HT29 and NIH3T3 cells during 24 h with dihydroethidium with increasing amounts of MAG. (**C**) GSH production was assessed during 6 h after incubation of CT26, HT29 and NIH3T3 cell during 24 h with monochlorobimane with increasing amounts of MAG. Data from at least three independent experiments have been pooled and were expressed as means ± SD of triplicates. ^*^p < 0.05; ^**^p < 0.01; ^***^p < 0.001 *versus* normal conditions.

To evaluate the modulation of ROS production by MAG, we measured H_2_O_2_, O_2_^−^ and GSH production by spectrofluorimetric assays using DiHydroEthidium (DHE), 2’,7’ DiChlorodihydroFluorescein DiAcetate (H_2_-DCFDA), and monochlorobimane through incubation of different cell lines with increasing amounts of MAG in the range of 0-25μM. H_2_O_2_ production was dose-dependently significantly increased with MAG in all cell lines (Figure 2A). At 15 μM of MAG, the increase in H_2_O_2_ was + 1382%, +1006%, +453% in CT26, HT29 and NIH3T3 cells, respectively. There were no significant differences in the production of O_2_^−^ in all cell lines using DHE signature as a specific marker (Figure 2B). In all cell lines, MAG decreased GSH production dose-dependently. At 15 μM of MAG, GSH production was decreased by 29%, 31%, 41% in CT26, HT29 and NIH3T3 cells, respectively (Figure 2C).

### MAG alone exerts differential cell-type cytotoxicity at 24 h

**Figure 3:**
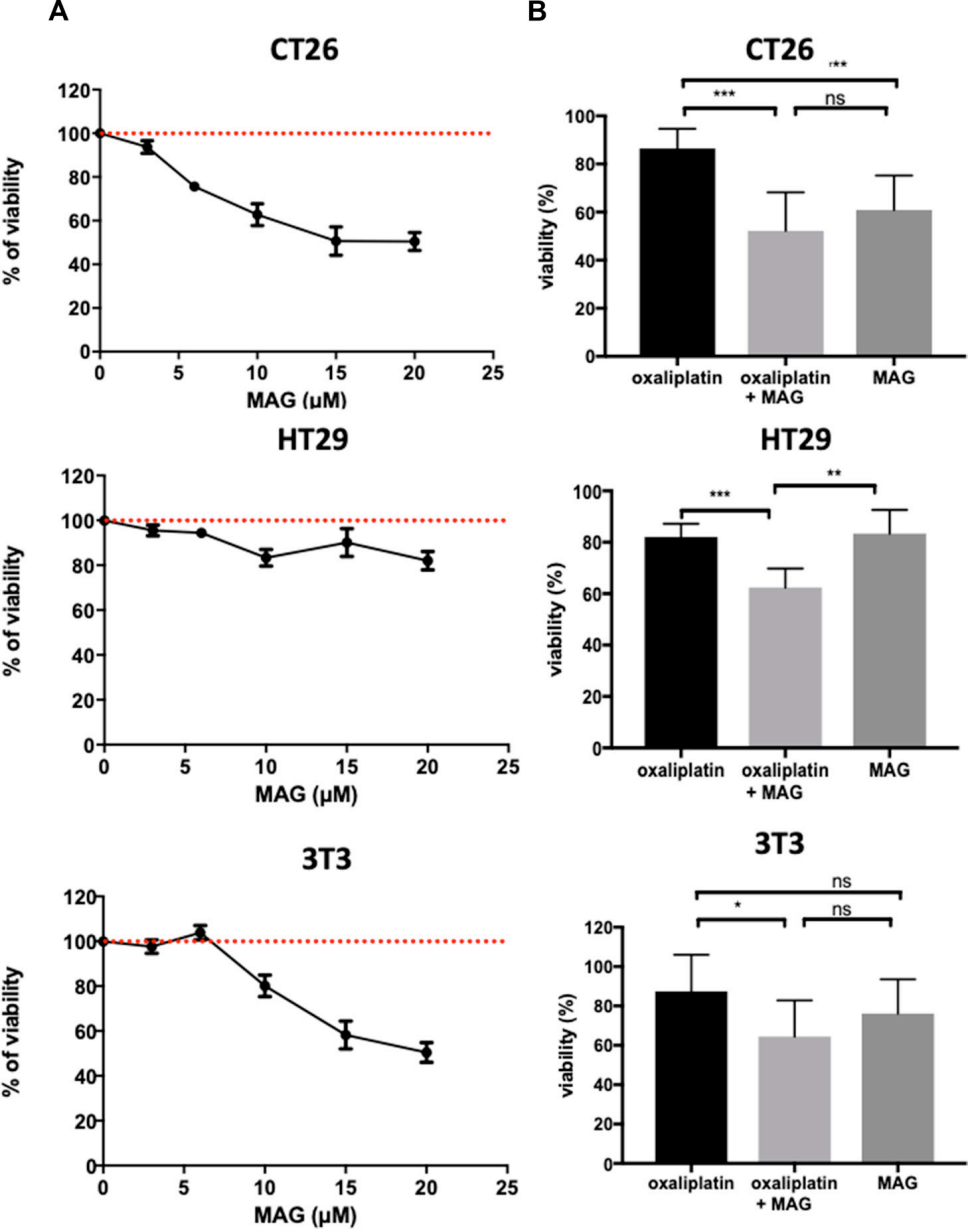
The cell-type cytotoxicity with MAG alone or in association with oxaliplatin. Viability was measured by crystal violet in CT26, HT29 and NIH3T3 cells at 24 h (**A**) with increasing amounts of MAG, and (**B**) with 10 μM of MAG and 5 μM of oxaliplatin. Data from at least three independent experiments have been pooled and were expressed as percent ± SD *versus* cells in culture medium alone (100% viability). ^*^p < 0.05; ^**^p < 0.01; ^***^p < 0.001 *versus* normal conditions.

To assess the antitumoral potential of MAG, we used cellular models involving fibroblasts and cancer cells. MAG displayed greater cytotoxicity in murine colon cancer cell lines (CT26) than in murine fibroblasts (NIH3T3). At 10 μM, MAG decreased the viability of CT26 cells by 39 ± 5% while it decreased the viability of NIH3T3 cells by only 24 ± 6%. The viability of human colon cancer cell line HT29 was slightly decreased by 17 ± 4% (Figure 3A).

### MAG exerts differential cell-type and additive cytotoxicity in association with oxaliplatin through the potentiation of an oxidative burst

To assess differential cell-type and additive cytotoxicity in association with oxaliplatin, we incubated CT26, HT29 and NIH3T3 cells with 10 μM MAG and 5 μM oxaliplatin. An additive cytotoxicity was observed when MAG was added in all the cell lines studied, compared to oxaliplatin alone. The viability of CT26, HT29 and NIH3T3 cells with oxaliplatin alone were 86.5 ± 2.7%, 82 ± 1.7%, 87.5 ± 6.1%, whereas the viability of CT26, HT29 and NIH3T3 cells with the association was significantly decreased to 52.2 ± 5.3%, 63.3 ± 3% and 64.4 ± 6% (*p* < 0.001, *p <* 0.001, and *p =* 0.02, respectively; Figure 3B).

MAG in equimolar association with oxaliplatin exerted a synergistic effect to trigger H_2_O_2_ production when compared to oxaliplatin alone in all cell lines. The oxidative burst was higher in CT26 and HT29 than in normal NIH3T3 cells (+1525%, +466% and +315%, respectively; Supplementary Figure 1A).

Under basal conditions, we observed an imbalance in GSH metabolism in murine tumor CT26 cells compared to murine normal NIH3T3 cells. Basal levels of GSH production were lower in CT26 than in NIH3T3 cells (6529 ± 759 and 14207 ± 689*p <* 0.0001; Figure 3B). In normal NIH3T3 cells, increasing the oxidative stress by incubation with oxaliplatin alone or associated with MAG increased intracellular GSH levels by 121% and 126%, respectively (Supplementary Figure 1B). Interestingly, incubating CT26 or HT29 colon cancer cells with oxaliplatin alone or in association with MAG failed to increase GSH synthesis (Supplementary Figure 1B).

### MAG exerts no pro-tumoral effect *in vivo* alone or in association with oxaliplatin

**Figure 4:**
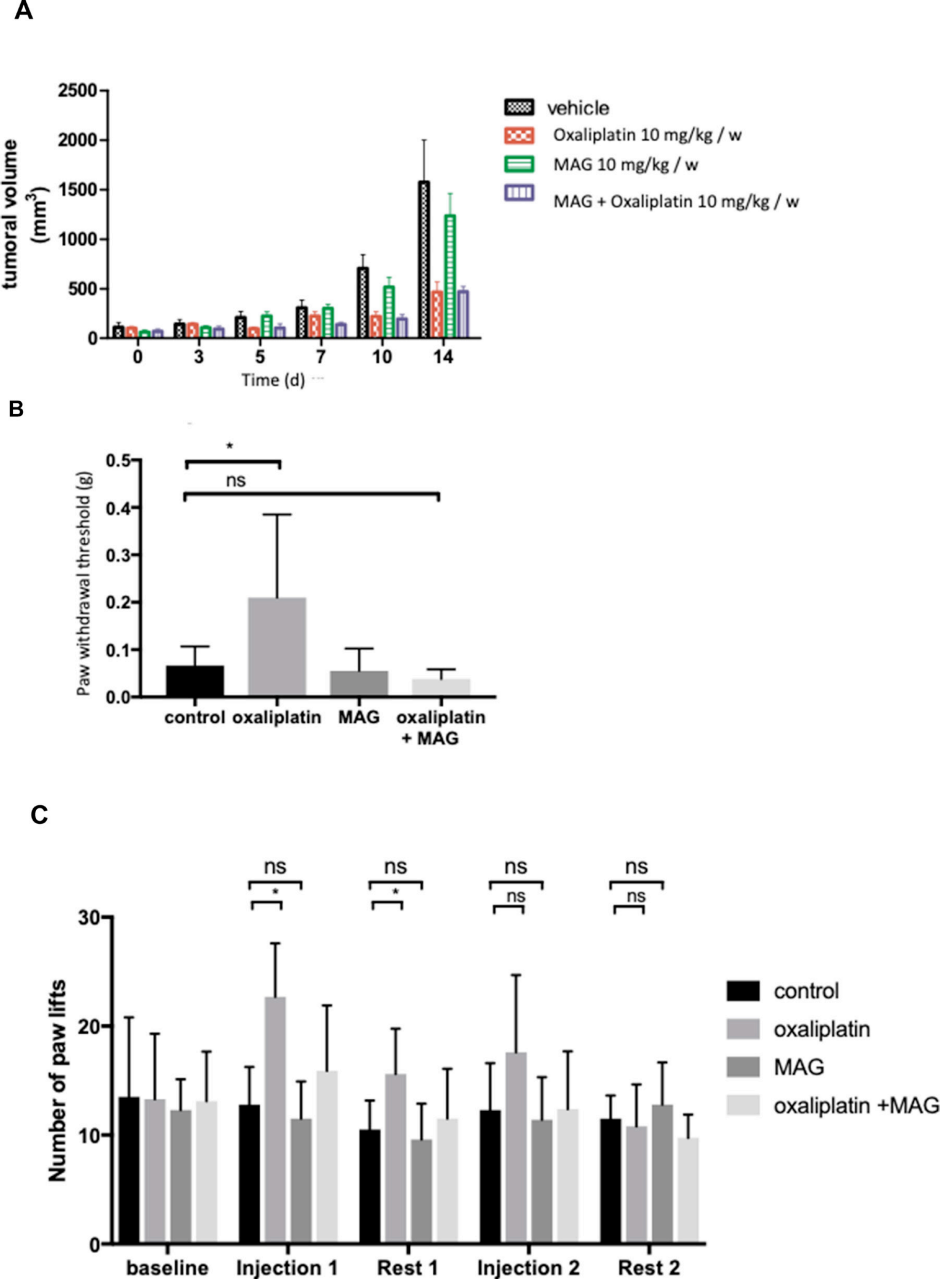
The *in vivo* effects of MAG associated with oxaliplatin on tumoral volume and on mouse tactile sensitivity and cold hyperalgesia. (**A**) Tumor growth following implantation of 2.10 [6] CT26 cells was measured in BALB/c mice treated by vehicle, oxaliplatin (10 mg/kg) and/or MAG (10 mg/kg) weekly for 15 days. (**B**) Experimental mice received oxaliplatin (10 mg/kg) and MAG (10 mg/kg) weekly for 8 weeks. Nociception was evaluated using a von Frey test at 8 weeks. (**C**) Evaluation of hyperalgesia required two 5-day cycles of daily oxaliplatin (3 mg/kg). Control mice received either oxaliplatin or vehicle alone. Cold hyperalgesia was evaluated using a cold plate set at 2° C. Data are expressed as the means ± SD of 13 and 6 different mice under each condition for the von Frey test and the cold plate test, respectively. ^*^p < 0.05; ^**^p < 0.01 *versus* vehicle or oxaliplatin.

Results obtained from the *in vitro* tests provided a rationale for the use of MAG as an antitumoral agent in association with oxaliplatin. From the day 10 of treatment on, tumors in mice treated with MAG alone were not statistically different in size than those in non-treated mice (516 ± 87 mm^3^ *vs* 705 ± 120 mm^3^ with vehicle). At the same day, tumors in mice treated with oxaliplatin or oxaliplatin with MAG were significantly decreased (respectively 202 ± 40 mm^3^ *vs* 705 ± 120 mm^3^, *p =* 0.004, 179 ± 35 mm^3^ *vs* 705 ± 120 mm^3^, *p =* 0.003). Oxaliplatin associated with MAG did not counteract oxaliplatin efficacy, since tumors in mice treated with oxaliplatin and MAG were not statistically different in sizes than those treated with oxaliplatin alone (Figure 4A).

### MAG diminishes oxaliplatin-induced acute cold hyperalgesia and chronic hypoesthesia

In the cold plate test, the mean number of brisk lifts of either hind paw was significantly higher in mice treated with oxaliplatin than in mice treated with vehicle at the end of cycle 1 and after 1 week of rest (Figure 4C). After weekly injections for 8 weeks, the Von Frey test showed that the threshold of withdrawal was higher in mice treated with oxaliplatin than in mice treated with vehicle alone (0.19 ± 0.17 g *vs* 0.067 ± 0.04 g, *p* = 0.04) (Figure 4B). These alterations in the cold plate test and in the Von Frey test were not observed in MAG plus oxaliplatin-treated animals. MAG protected acute cold hyperalgesia and chronic hypoesthesia when associated with oxaliplatin.

### MAG reduces oxaliplatin-induced neurosensitive alterations *in vivo*

Having observed the restoration of sensitive function of peripheral nerves in mice, we further studied the excitability properties of sensitive peripheral nerves of animals after 8 weeks of treatment, using *in vivo* electrophysiological recordings (Supplementary Figure 2). Oxaliplatin-treated mice (*n =* 5), compared to control animals (*n =* 5), showed a reduced maximal CNAP amplitude (0.021 ± 0.005 mV *vs* 0.037 ± 0.006 mV, *p* = 0.004), a decreased stimulus necessary to generate 50% maximal CNAP amplitude (0.08 ± 0.02 mA *vs* 0.21 ± 0.04 mA, *p =* 0.002), and an increased latency (3.52 ± 0.07 ms *vs* 3.08 ± 0.03 ms, *p =* 0.00003). These alterations of CNAP variables were much less marked in mice treated with both oxaliplatin and MAG (*n =* 5) and were not observed in MAG-treated animals (*n =* 5), as illustrated in Supplementary Figure 2. In particular, compared to oxaliplatin-treated animals, the CNAP recorded from mice administered with both MAG and oxaliplatin was partially restored with significantly higher stimulus necessary to generate 50% maximal CNAP amplitude and lower latency, *i. e.* 0.12 ± 0.02 mA (*p =* 0.022) and 3.19 ± 0.03 mA (*p =* 0.0001), respectively.

### MAG protects from oxaliplatin-induced neuromuscular alterations *in vivo*

It has been shown that oxaliplatin also alters excitability of peripheral nerve fibers. Neuromuscular excitability was assessed in all experimental mice groups after 4 weeks of treatment. Oxaliplatin mice presented alterations in neuromuscular excitability waveforms and associated variables, compared to mice injected with vehicle alone (Supplementary Table 1). First, oxaliplatin induced alterations in CMAP by significantly increasing the peak response and reducing the stimulus needed to evoke 50% of a maximal response, with no change in latency, *i. e.* no change in the neurotransmission. Second, it increased threshold changes in response to hyperpolarizing currents (threshold electrotonus), which could be explained by reduced density and/or functioning of inward rectifier potassium channels. Third, it reduced minimum and hyperpolarizing slopes of the current–threshold relationship, indicating decreased density and/or functioning of cyclic nucleotide-gated channels. Finally, oxaliplatin decreased the superexcitability period of recovery cycle, reflecting fast potassium channel modification. All these alterations were not detected in mice injected with oxaliplatin plus MAG or MAG alone (Supplementary Table 1).

### MAG prevents oxaliplatin-induced peripheral demyelination

**Figure 5:**
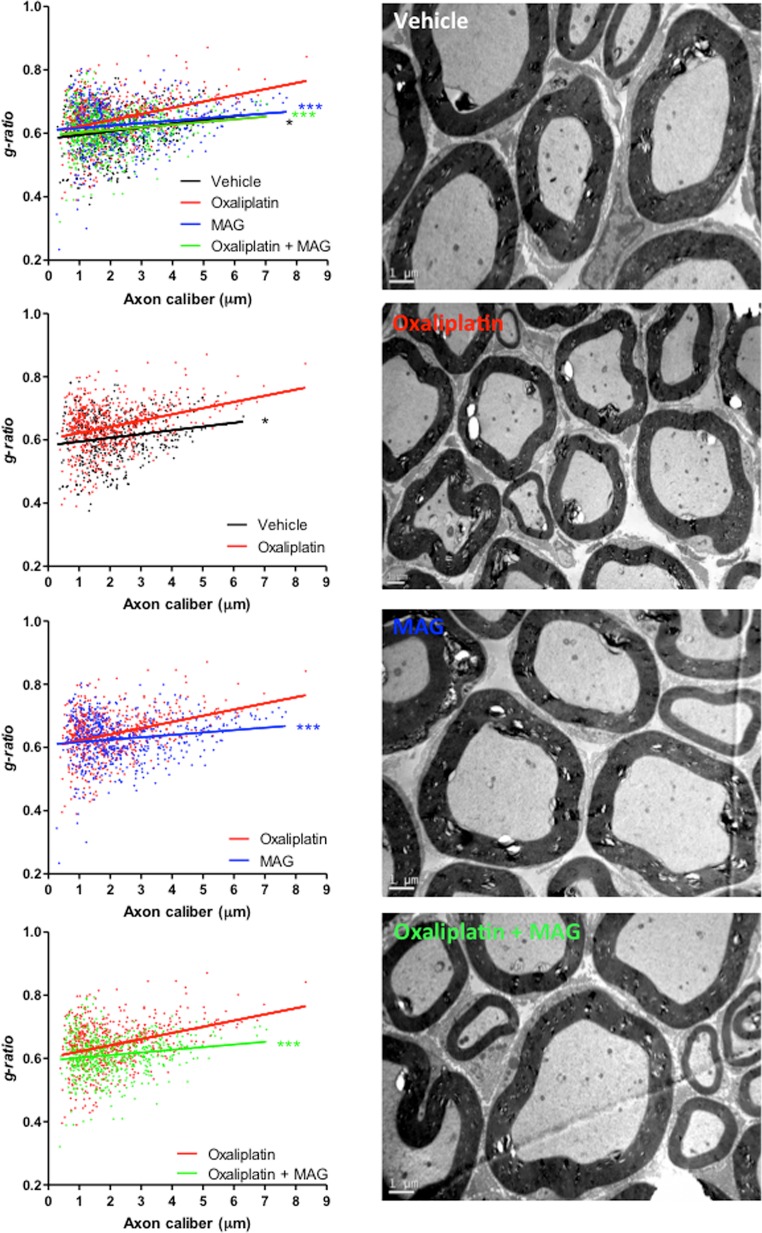
The EM micrographs of cross-sections of sciatic nerves from vehicle, oxaliplatin, MAG and oxaliplatin + MAG mice at 4 weeks. Representative images are shown. Scale bar, 1 μm. Quantification of myelin through g-ratio analysis reported to axonal caliber. Vehicle, *n =* 3; oxaliplatin, *n =* 3; MAG, *n =* 3; oxaliplatin + MAG, *n =* 3. At least 490 axons per group were analyzed. ^*^P < 0.05, ^**^P < 0.01, ^***^P < 0.001 linear regression test with slopes compared.

The myelin sheaths of sciatic nerves from experimental and control groups of mice were assessed. Analyses revealed a profound peripheral demyelination in oxaliplatin-treated mice compared to animals injected with vehicle, oxaliplatin plus MAG or MAG alone (Figure 5). Semi-automated computerized measurement of myelin thickness allowed the quantification of the profound demyelination in sciatic nerves from oxaliplatin mice (0.0196 ± 0.0023 with oxaliplatin *versus* 0.0119 ± 0.0031 with vehicle, *p =* 0.0423) (Figure 5). Associating MAG to the chemotherapy abrogated the reduction in myelin sheath thickness observed in oxaliplatin mice (0.0084 ± 0.0022 with oxaliplatin plus MAG *versus* 0.0119 ± 0.0031 with vehicle, *p =* 0.3469 and *versus* 0.0196 ± 0.0023 with oxaliplatin alone, *p =* 0.0005) (Figure 5). MAG did not impair myelin formation, nor did it lead to excessive myelination (0.0119 ± 0.0031 with vehicle *versus* 0.0076 ± 0.0021 with MAG, *p =* 0.2444).

## DISCUSSION

Our results highlighted that MAG presented a SOD modulator effect by increasing H_2_O_2_ levels *in vitro* and reduced oxaliplatin-induced neurotoxicity *in vivo*. MAG did not modify the oxaliplatin’s antitumoral efficacy *in vivo*. All methods used in this report were consistent, either at a cellular, histological, or functional level.

MAG, based on the anti-oxidant properties was first explored in a cancer model to evaluate its antitumoral effect. *In vitro*, this report evidenced the differential cytotoxic effect of MAG in tumoral cells compared to normal cells. These results are in line with the ROS modulation activity of MAG. The differential effects of MAG in normal neuronal cells and cancer cells may be related to the higher basal levels of ROS in cancer cells compared to normal cells [7, 11, 31]. Indeed, we have confirmed here that the basal concentration of H_2_O_2_ was higher in cancer cells than in normal cells. It has been previously related to a higher rate of production of superoxide anions (O2•−) [3, 7, 31]. MAG enhances the generation of H_2_O_2_ in cancer cells, while the threshold of toxicity of H_2_O_2_ is reached more easily in cancer cells than in normal cells. The lower levels of antioxidant enzymes such as catalase, glutathione peroxidase, reductase and their respective cofactors in cancer cells compared to normal cells amplify this phenomenon [7, 11, 31, 32]. Cancer cells are no longer able to detoxify H_2_O_2_ in H_2_O when antioxidant enzymes are overtaken by high levels of H_2_O_2_.

On the other hand, in normal cells, MAG limits O2•− toxic mitochondrial effect by the dismutation of O_2_ in H_2_O_2_. Then, through the catalase activity of MAG already mentioned in another report [25] and the higher basal levels of GSH that we found in normal cells, H_2_O_2_ is detoxified in H_2_O.

In this report we failed to show a variation in anion superoxide production using DHE assay, although we could expect a significant decrease in superoxide with increasing amounts of MAG. Due to its short lifetime and rapid reaction with cellular components including the dismutation in hydrogen peroxide catalyzed by superoxide dismutase (SOD), anion superoxide remains hard to detect and quantify in cellular systems [33]. The detection at 1 hour instead of 24 hours might be a better time to detect anion superoxide. It also has been shown that although the measured amount of 2-hydroxyethidium is a good index of superoxide anion, it cannot be directly equated to the value of its intracellular flux [34]. Despite these limitations, the increase in H_2_O_2_ production was consistent with the occurrence of superoxide dismutation in this article. Our findings are in line with other reports using MAG in other models of oxidative stress [25, 26]. A limitation of our study is that the protective effect of MAG on normal cells was only studied on fibroblast cells. Preferred cells would have been neuron cells from dorsal root ganglia. Nevertheless, the half-life and the low rate of production of neurons cells do not allow the evaluation of MAG for its neuroprotective effect in such conditions. Furthermore, we found that MAG exerts additive cytotoxicity when associated with oxaliplatin. Indeed, MAG potentiated the oxidative burst induced by oxaliplatin by increasing H_2_O_2_ production. Surprisingly, the cytotoxic effect of MAG observed *in vivo* was not identified in our murine model despite the confirmed efficacy of oxaliplatin.

Based on the protective effect of MAG on normal cells *in vitro*, this compound was explored to evaluate its neuroprotective effect when associated with oxaliplatin. Our findings reported the beneficial effects of MAG on oxaliplatin-induced peripheral neuropathies. Using standardized functional tests, we evaluated the different manifestations of oxaliplatin-induced neuropathies. Acute neuropathy is defined by hyperalgesia, pseudolaryngospasm, muscle spasms triggered by cold and chronic oxaliplatin neuropathy by bilaterally distal symmetrical sensory symptoms (e. g., numbness, tingling, and pain) [35–37] and ultimately hypoesthesia. Although the acute form can be quite difficult, it is the chronic CIPN that is the general dose-limiting problem and the main cause of complete discontinuation of oxaliplatin treatment. Two different tests were used to mimic the different patterns: acute neurotoxicity was evaluated by cold plate tests and chronic neuropathy by Von Frey tests, as previously described [11, 38]. MAG prevented neurotoxic effects in both of these tests. The mechanisms underlying acute and chronic oxaliplatin-induced neuropathies are multifactorial and quite complex. Several pathways involving multiple key proteins are at play in the development of neurotoxicity: mitochondrial dysfunction including direct mitochondrial DNA (mtDNA) damage, dysregulation of calcium homoeostasis, ROS modulation, modulation of ion channels expression, inflammation, axonal damage and demyelination [9, 39, 40].

In our study, we identified that MAG might prevent acute and chronic neuropathies through ROS modulation. In acute hyperalgesia induced by oxaliplatin, the sensitization to pain thresholds in the dorsal root ganglia neuron has been linked to ROS modulation [41]. Similar results were confirmed by the use of ROS scavengers molecules to block sensitization to pain in a murine model of neuropathic pain [42]. Detoxification of H_2_O_2_ through modulation of GSH has been described as a potential key to reduce chronic neuropathy, either in chronic diabetes-induced neuropathy [43] or in oxaliplatin-induced neuropathy [44]. In addition, another key mechanism that could explain the neuroprotective effect of MAG is its anti-inflammatory properties as reported by Mathieu *et al* [26]. Indeed, pro-inflammatory cytokines are involved in the development of peripheral neuropathies in mice and humans [45–47], and MAG may protect nerve fibers by reducing local inflammation mediated by oxidative species. Meantime, the results gathered by electrophysiological studies indicate that the mechanisms at play in MAG-mediated neuroprotection also involve the modulation of either the functionality or the density of ion channels. Oxaliplatin can indeed impact several types of ion channels, namely transitory sodium channels, inward-rectifying potassium channels and cyclic nucleotide–gated ion channels [11, 48]. Calcium is a pre-requisite for normal functioning of cyclic nucleotide-gated and manganese has been shown to block calcium flux through several ion channels [49]. It is possible that by blocking calcium-mediated cyclic nucleotide-gated signaling, the chelate of manganese MAG might up-regulate compensatory signaling pathways through enhanced functioning of ion channels such as transitory sodium channels and inward-rectifying potassium channels incriminated in oxaliplatin neuropathies. As calcium can also be involved in cognitive performance [50], it would be interesting to investigate the effect of MAG on the central nervous system, *i. e.* the cognitive decline of oxaliplatin-treated patients [51], and on chemobrain processes. Morphological data obtained by electron microscopy are in line with previous reports of profound oxaliplatin-induced demyelination [39, 52]. When administered with oxaliplatin, MAG rescued this destruction of the peripheral myelin. Mice only receiving MAG did not present lower G-ratios than mice that received only the vehicle. Therefore, MAG did not induce a hypermyelination, a process which has also been shown to be deleterious for the nervous system [53, 54]. This observation indicates that MAG may exert its neuroprotective properties by preventing the destruction of the myelin rather than through a remyelinating process resulting from a compensatory production of myelin. Based on all these potential mechanisms, all SOD modulators might have a neuroprotective effect as identified in our study using MAG.

## MATERIALS AND METHODS

### Cell culture and treatments

All cell lines were authenticated by short tandem repeat analysis and routinely tested for mycoplasma. All cell lines were purchased from the ATCC. CT26 (mouse colon carcinoma) and NIH3T3 (mouse embryonic fibroblast cells) cells were grown in Dulbecco’s Modified Eagle Medium with 10% FBS, penicillin/streptomycin, and L-glutamine supplementation. HT29 (Human colon carcinoma) cells were grown in McCoy’s 5A Modified Medium with 10% FBS, penicillin/streptomycin, and L-glutamine supplementation.

### ROS, GSH and viability assays

All cells (1.5 × 10^4^ - 10^5^ cells per well) were seeded in 96-well plates (Corning). After 24 hours, they were incubated for 18 hours with 0 to 50 μM of MAG (Sigma-Aldrich) and/or treated with 5 μM of oxaliplatin (Accord Healthcare Limited).

Cell viability was assessed by crystal violet assay, and results were expressed as the mean percentage of viable cells ± SD *versus* cells not exposed to oxaliplatin nor MAG (100% viability).

Cellular production of hydrogen peroxide (H_2_O_2_), reduced glutathione (GSH) and hydroxyl radical O_2_^−^ were assessed by spectrofluorimetry with 2’,7’ DiChlorodihydroFluorescein DiAcetate (H_2_-DCFDA), monochlorobimane and DiHydroEthidium (DHE), respectively. Cells were washed in Phosphate Buffer Saline (PBS) and incubated with 100 μL per well of 5 μM H_2_-DCFDA, 15 μM DHE and 5 μM monochlorobimane, diluted in PBS (Molecular Probes). H_2_O_2_ levels were assayed by spectrofluorimetry on a fusion spectrofluorimeter (PackardBell). Fluorescence intensity was recorded every hour over a period of 5 hours. The number of cells was evaluated by crystal violet assay. Levels of H_2_O_2_, O_2_^−^ and GSH were calculated in each sample as follows: reactive oxygen species rate (arbitrary units min^-1^ 10^5^ cells^-1^) = [fluorescence intensity (arbitrary units) at T300 min − fluorescence intensity (arbitrary units) at T0] per 60 min per number of cells as measured by crystal violet assay. Student *t* test were performed.

### Animals

BALB/c female mice at 6 weeks of age were purchased (Janvier Laboratory). All animals were housed in ventilated cages (*n =* 7 to 10 animals per cage) with rodent diet (Teklad global 16% protein, Envigo S. A. R. L, 03800 GANNAT, France) and water ad libitum and were exposed to a standard light cycle of 12 hours on and 12 hours off. Mice were euthanized by cervical dislocation under the inhalation anesthesia with 2.5-3.5% isoflurane with O_2_. All experiments were performed in accordance with European and French institutional guidelines (Directive 2010/63/EU, Ethics committee CEEA 34 – APAFIS authorization #8394).

### Ethics statement

All animal experiments were carried out in compliance with French institutional guidelines (Directive 2010/63/EU) and approved by the Animal Experimentation and Ethics Committee of Paris Descartes University (Ethics committee CEEA 34 – APAFIS authorization #8394).

All efforts were pursued to minimize animal distress and to reduce the number of animals used.

### Mice model for behavioral studies

We evaluated neurotoxicity in a mice model by sensorial tests with two therapeutic schedules of injections to mimic acute and chronic development of oxaliplatin-induced neuropathy. Chronic neuropathy is characterized by hypoesthesia and motor dysfunction following long-term treatment by oxaliplatin and can be assessed by the Von Frey test. The model used to obtain chronic neurotoxicity was based on weekly administration during a prolonged period (more than 4 weeks). For the Von Frey test, mice (*n* = 8 per group) received weekly direct intraperitoneal injections (200 μL) on Wednesdays at 9 a. m. of, either oxaliplatin (10 mg/kg) immediately followed by vehicle, MAG (10 mg/kg) immediately followed by vehicle, oxaliplatin immediately followed by MAG, or vehicle, for 8 weeks. The Von Frey test was repeated 6 days after the injection on Tuesday afternoons. The week before the experiments, mice were familiarized with the Von Frey test procedure. The principle of this test is standardized: once the mouse is calm and motionless (after 5 minutes), a hind paw is touched with the tip of a flexible fiber of given length and diameter. The fiber is pressed against the plantar surface at a right angle and exerts a vertical force. The force increases until the fiber bends. After the fiber bends, and even if the investigator continues to push the probe, there is no more force of application but more bend. This principle makes it possible for the investigator to apply a reproducible force to the skin surface. Rodents exhibit a paw withdrawal reflex as soon as the paw is touched. The Von Frey test was considered positive when the animal indicated a perception of the fiber by pulling back its paw [27]. The Von Frey filaments kit included filaments eliciting a bending force from 0.008 to 300g. Our tests performed in mice identified modifications with filaments with bending forces as follow: 0.008-0.02-0.04-0.07-0.16-0.4-0.6. Paw movements associated with locomotion were not counted as a withdrawal response. Each group was blinded for the investigator. The control group (intraperitoneal injection of vehicle) was considered as reference for normal Von Frey value.

Acute neuropathy is characterized by hyperalgesia/allodynia upon exposure to cold and can be evaluated by the cold plate test. Cold sensitivity was evaluated using the protocol described by Ta *et al* [28], after high daily dose of treatment. Cold sensitivity was evaluated by the cold-plate assay at the optimal temperature of 2° C ± 0.2° C. Mice were treated daily either with direct intraperitoneal injection of oxaliplatin (3 mg/kg) immediately followed by vehicle, MAG immediately followed by vehicle (3 mg/kg), oxaliplatin immediately followed by MAG (3 mg/kg each) or vehicle for 5 days, followed by 5 days of rest, for 2 cycles. The week prior to the experiments, mice were familiarized with the cold plate test procedure. The total number of brisk lifts of one or the other hind paw in 5 minutes was counted as the response to cold sensitivity, and the same observer was tasked with counting to ensure accuracy. We excluded brisk lifts involved in normal locomotion defined as coordinated movements of all four limbs. A cold-plate test was repeated at baseline before the treatment, at day 5 of treatment, and after drug treatment at day 10 of each of the two cycles. Results were expressed as the mean ± SD of the observers’ counts. Mann Whitney tests were performed.

### Mice model and electrophysiology

Mice were treated for 4 weeks with direct intraperitoneal injections (200 μL) of vehicle (*n =* 9), oxaliplatin immediately followed by vehicle (*n =* 7), oxaliplatin immediately followed by MAG (*n =* 9) or MAG alone immediately followed by vehicle (*n =* 8). At day 28, the sensory and/or neuromuscular excitability was assessed *in vivo* on mice under 2.5% isoflurane (AErrane^®^) anesthesia with O2 by minimally invasive electrophysiological methods, as previously described [29]. Each group was blinded for the investigator. For sensory excitability exploration, the compound nerve action potential (CNAP) was recorded using needle electrodes inserted into the base of the mouse’s tail, in response to stimulation of the caudal nerve applied at the distal part of the tail by means of surface electrodes. The mouse was submitted to the first session of excitability measurements (TRONDE protocol) to establish the stimulus-response relationship and thus evaluate notably the CNAP maximal amplitude, the stimulation intensity that had to be applied to evoke a CNAP of 50% maximal amplitude and the latency measured from stimulation onset to peak amplitude, giving information on the global sensory excitability state. For neuromuscular excitability exploration, electrical stimulation was delivered to the sciatic motor nerve by means of surface electrodes, and the compound muscle action potential (CMAP) was recorded through needle electrodes inserted into the plantar muscle. The mouse was submitted to five sessions of excitability measurements (TRONDE protocol) which consisted in five different excitability tests performed together: (1) the stimulus-response relationship, (2) the current-threshold relationship, (3) the strength-duration relationship, (4) the threshold electrotonus, and (5) the recovery cycle. The thirty variables determined by these excitability tests were analyzed in order to get information on the density and functional status of ion channels, receptors and pumps, as well as on the passive membrane properties of the neuromuscular system. Student *t* tests were performed.

### Mice model for tumor growth evaluation

We injected subcutaneously 1.000,000 viable CT26 cells suspended in DMEM into the back of BALB/c mice. After tumor growth of 200 to 500 mm^3^, animals (*n =* 8 per group) were randomized and received weekly direct intraperitoneal injections of oxaliplatin (10 mg/kg) immediately followed by vehicle, MAG (10 mg/kg) immediately followed by vehicle, oxaliplatin immediately followed by MAG (10 mg/kg each), or vehicle. Following randomization, tumor size was measured with a numeric caliper twice a week for 3 weeks. Each group was blinded for the investigator. Sacrifice occurred after a threshold of a maximum tumor size in order to comply with ethical guidelines. Tumor volume was calculated as follows: TV (mm^3^) = (L × W^2^)/2, where L is the longest and W the shortest radius of the tumor in millimeters. Results were expressed as means ± SD of tumor volumes (*n =* 10 in each group). Data were analyzed with the Cox model.

### Electron microscopy morphological study of mouse sciatic nerves

Mice (*n =* 3 per groups) received weekly injections of oxaliplatin (10 mg/kg) and vehicle, MAG (10 mg/kg) and vehicle, oxaliplatin and MAG, or vehicle for 4 weeks. Animals were anesthetized by intraperitoneal injections of 100 mg/kg ketamine and 10 mg/kg xylazine instead of isoflurane anesthesia and then intracardially perfused with first, 0.1 M phosphate buffer, pH 7.4, for 8 minutes and then 4% paraformaldehyde, 2.5% glutaraldehyde, and 0.1 M phosphate buffer, pH 7.4. Tissues were dissected 24 hours later and immersed in the fixative solution at 4° C for 72 hours, washed in phosphate buffer, post-fixed in 2% osmium tetroxide, dehydrated in graded ethanol, and embedded in epoxy resin. Ultrathin sections (50–90 nm) were cut on an ultramicrotome (8800 Ultrotome III; LKB Bromma) and collected on 300-mesh nickel grids. Staining was performed on drops of 4% aqueous uranyl acetate, followed by Reynolds lead citrate. Ultrastructural analyses were performed with a JEOL JEM-1011 electron microscope and digitalized with the DigitalMicrograph software. G-ratios and diameters were calculated with the ImageJ software (National Institutes of Health) and the plugin *g*-ratio version 3.2 (available online at http://gratio.efil.de) according to previously published material [30]. G-ratios were calculated from enclosed areas, excluding equivocal artefactual degradation of axons, and considering first, the diameter of the axon without the myelin and dividing it by the diameter of the axon plus the myelin sheaths surrounding it. The plugin allows a semi-automated analysis of randomly selected fibers from the enclosed area with both diameters being considered and automatically processed through the algorithm to calculate the G-ratio. A minimum of 490 randomly selected axons were analyzed per experimental group, with at least 3 mice per group.

### Statistical analysis

Statistical analyses were performed using GraphPad Prism 5. Artwork was also created using GraphPad Prism 5, except for the electrophysiological study artwork which was created using the Qtrac© software. Student t test or Mann-Whitney *U* test, depending on the equality of variances estimated using Lilliefors test were used as noted and Cox model were performed. The *p* values are denoted as follows: ^*^p < 0.05; ^**^p < 0.01; ^***^p < 0.001; ^****^p < 0.0001.

## CONCLUSIONS

*In vitro* MAG, a Mn superoxide dismutase mimic, increases levels of hydrogen peroxide by the detoxification of anion peroxide. MAG has additive and differential cell-type cytotoxicity when combined with oxaliplatin. Normal cells, though an effective anti-oxidant glutathione system, are more able to take charge of hydrogen peroxide than tumoral cells.

*In vivo,* MAG prevents the appearance of sensitive axonal neuropathy and neuromuscular dysfunction induced by oxaliplatin without affecting its antitumor action. Mechanisms at play in MAG-neuroprotection are multiple, including the modulation of ROS, the modulation of ion channels expression, the protection of axons and myelin sheath. These *in vitro* and *in vivo* data advocate the development of a molecule chemically associating MAG and oxaliplatin. Similarly, a clinical phase I study using MAG in patients receiving oxaliplatin-based chemotherapy should be performed to confirm our *in vitro* and *in vivo* results.

## SUPPLEMENTARY MATERIALS


