# Supplementary material for: Oxaliplatin-induced neuropathy: the preventive effect of a new super-oxide dismutase modulator
Source: Oncotarget. 2019 Nov 5;10(60):6418–31. doi: 10.18632/oncotarget.27248 (PMC6849645; doi:10.18632/oncotarget.27248)
Supplement: Supplementary file 1 [file oncotarget-10-6418-s001.pdf]

Oxaliplatin-induced neuropathy: the preventive effect of a new super-oxide dismutase modulator

SUPPLEMENTARY MATERIALS

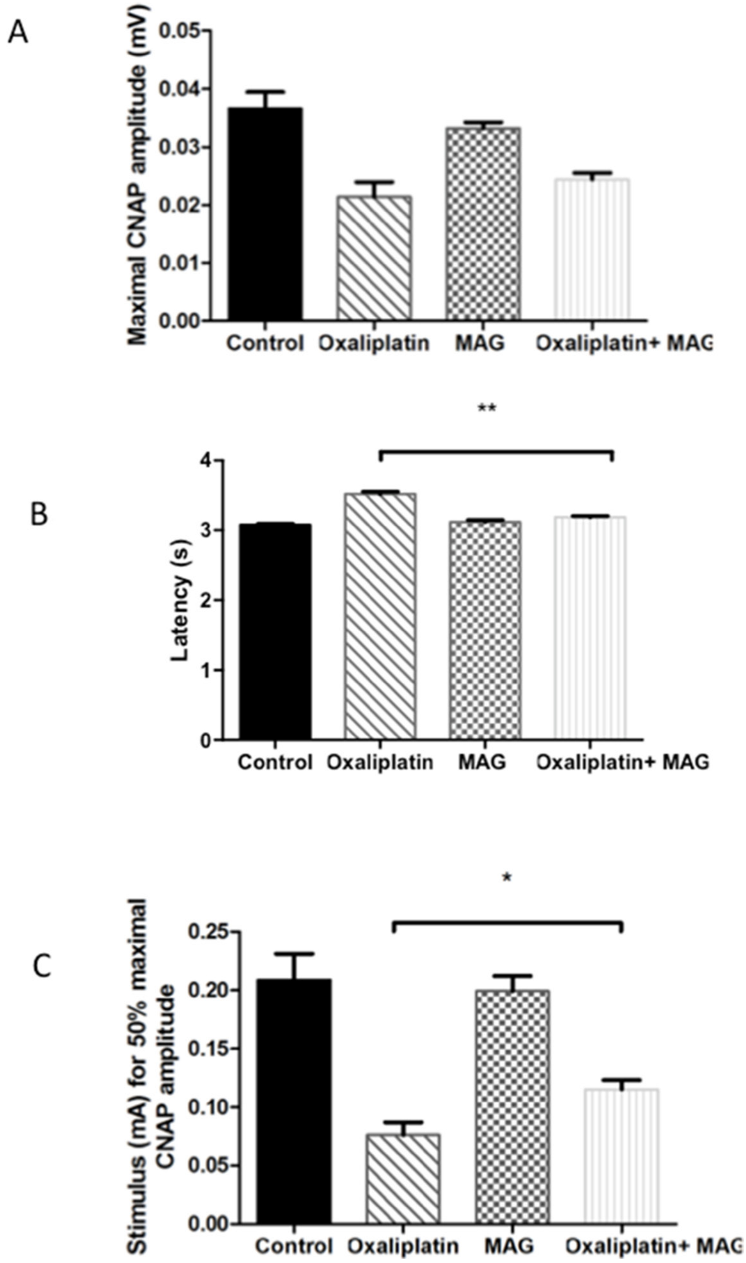

Supplementary Figure 1: The *in vitro* production of MAG associated with oxaliplatin-induced ROS production in CT26, HT29 and NIH3T3 cells at 24 h. (**A**) Hydrogen peroxide production was assessed during 6 h after incubation of CT26, HT29 and NIH3T3 cells during 24 h with 2',7' dichlorodihydrofluorescein diacetate with 10 µM of MAG and 5 µM of oxaliplatin. (**B**) GSH production was assessed during 6 h after incubation of CT26, HT29 and NIH3T3 cells during 24 h with monochlorobimane with 10 µM of MAG and 5 µM of oxaliplatin. Data from at least three independent experiments have been pooled and were expressed as means ± SD of triplicates. $^{*}p < 0.05$; $^{**}p < 0.01$; $^{***}p < 0.001$ *versus* normal conditions.

A

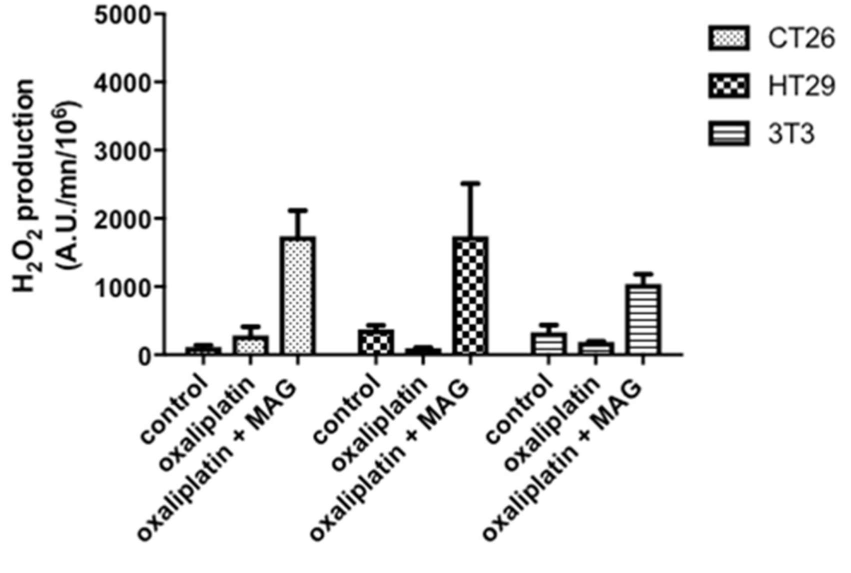

B

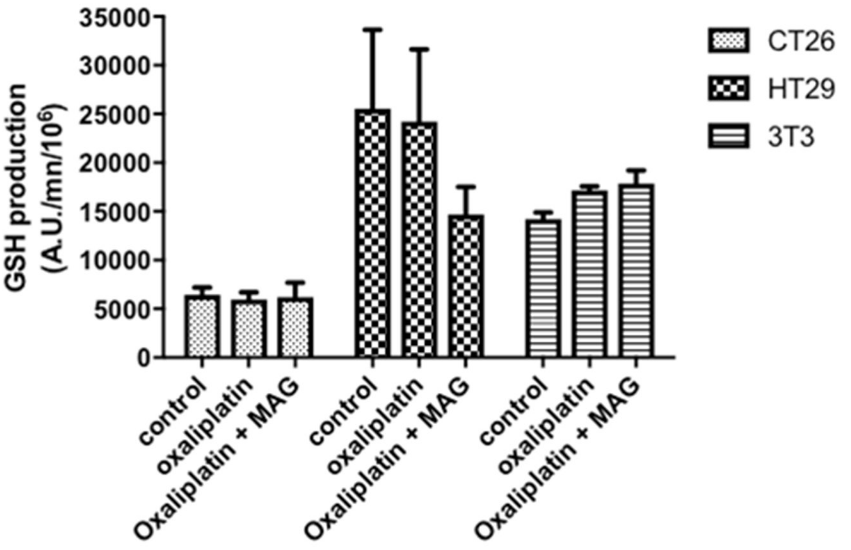

Supplementary Figure 2: The *in vivo* effects of oxaliplatin and MAG on mouse sensory excitability variables. Histograms of mean values ± SD of maximal CNAP peak amplitude (upper), stimulus intensity necessary to evoke 50% of maximal CNAP amplitude (middle), and latency (lower), determined from recordings at the tail base in response to caudal nerve stimulation in mice treated for 8 weeks with vehicle (n = 5), oxaliplatin (n = 5), oxaliplatin and MAG (n = 5) or MAG alone (n = 5). $^{*}p < 0.05$; $^{**}p < 0.01$ *versus* oxaliplatin.

Supplementary Table 1: The comparison of neuromuscular excitability variables

Derived excitability variables	Vehicle	Oxaliplatin	Oxaliplatin + MAG	MAG
**06.** Peak re sponse (mV)	3.419 ± 0.450	4.196 ± 0.380 *	3.483 ± 0.190	3.555 ± 0.220
**19.** Lat ency (ms)	3.508 ± 0.427	3.385 ± 0.235	3.719 ± 0.144	3.646 ± 0.262
**01.** Stimu lus (mA) for 50% max response	0.213 ± 0.040	0.138 ± 0.070 *	0.234 ± 0.045	0.242 ± 0.052
**05.** Stimu lus-response slope	3.846 ± 0.220	3.799 ± 0.190	3.695 ± 0.480	3.633 ± 0.250
A **03.** St ren gt h-duration time constant (ms)	0.4268 ± 0.0296	0.4683 ± 0.0389	0.3066 ± 0.0333	0.3439 ± 0.0483
A **4.** Rhe obase (mA)	0.1176 ± 0.0800	0.1154 ± 0.0800	0.1290 ± 0.0600	0.1219 ± 0.0700
B **12.** TE d (10–20 ms) / 40%	53.30 ± 1.98	53.70 ± 3.64	48.81 ± 1.97	49.06 ± 2.28
B **25.** TE d (peak) / 40%	54.14 ± 1.85	54.39 ± 3.12	49.19 ± 2.00	48.90 ± 2.26
B **35.** TE d (peak) / 20%	32.67 ± 1.49	32.46 ± 2.55	28.32 ± 1.37	28.32 ± 1.77
B **20.** TE d (40–60 ms) / 40%	38.58 ± 1.72	40.09 ± 2.25	35.48 ± 1.16	37.34 ± 1.91
B **21.** TE d (90–100 ms) / 40%	33.55 ± 1.61	35.74 ± 2.21	30.63 ± 1.17	32.39 ± 2.33
B **7.** Acco m modation half-time (ms) / 40%	31.32 ± 1.23	30.43 ± 1.25	35.18 ± 2.32	35.96 ± 1.82
B **26.** S2 a cc ommodation / 40%	20.60 ± 1.15	18.65 ± 1.81	16.47 ± 1.08	16.51 ± 1.90
B **23.** TE d (undershoot) / 40%	−15.86 ± 0.90	−15.04 ± 2.27	−13.38 ± 0.77	−13.90 ± 1.59
B **22.** TE h (10–20 ms) / −40%	−79.87 ± 2.43	−84.48 ± 2.75	−73.91 ± 3.03	−74.22 ± 4.00
B **30.** TE h (20–40 ms) / −40%	−92.60 ± 3.63	−101.30 ± 4.32	−86.50 ± 4.16	−88.66 ± 4.71
B **11.** TE h (90–100 ms) / −40%	−93.84 ± 7.21	−120.80 ± 4.79 *	−98.16 ± 6.28	−101.50 ± 8.67
B **31.** TE h (s lope 101-140 ms) / −40%	1.42 ± 0.22	2.05 ± 0.08 *	1.66 ± 0.17	1.68 ± 0.24
B **37.** TEh (peak) / −70%	−205.80 ± 13.00	−246.20 ± 6.87 *	−219.80 ± 11.20	−215.80 ± 13.90
B **38.** S3 / −70%	22.66 ± 5.84	39.65 ± 3.73 **	25.29 ± 9.41	26.04 ± 4.28
B **24.** TE h (overshoot) / -40%	13.23 ± 1.01	14.05 ± 1.55	11.67 ± 0.93	10.93 ± 0.78
C **07.** Rest i n g slope	0.887 ± 0.084	0.880 ± 0.025	0.900 ± 0.062	0.812 ± 0.060
C **08.** Min imum slope	0.311 ± 0.023	0.277 ± 0.016 *	0.314 ± 0.038	0.284 ± 0.022
C **28**. H yp erpolarizing slope	0.947 ± 0.042	0.467 ± 0.020 **	1.044 ± 0.266	1.030 ± 0.360
D **32.** Ref ra ctoriness at 2 ms (%)	37.23 ± 7.78	34.56 ± 8.96	40.84 ± 4.88	44.99 ± 8.09
D **29.** Ref ra ctoriness at 2.5 ms (%)	28.34 ± 3.83	30.39 ± 4.70	27.63 ± 3.01	26.30 ± 4.04
D **13.** Su pe rexcitability (%)	−24.82 ± 8.29	−14.68 ± 3.22 *	−24.21 ± 0.92	−5.59 ± 1.25
D **34.** Su pe rexcitability at 5 ms (%)	−4.42 ± 2.09	1.46 ± 1.89 *	−2.26 ± 0.10	−4.12 ± 1.02
D **3.** Su pe rexcitability at 7 ms (%)	−5.52 ± 1.42	−1.08 ± 1.44 *	−4.05 ± 0.89	−5.75 ± 1.07
D **14.** Su be xcitability (%)	7.41 ± 0.74	6.30 ± 0.70	6.35 ± 0.30	6.33 ± 0.47

Derived excitability variables (means ± SD) from plantar muscle recordings in response to motor sciatic nerve stimulation of mice treated for 4 weeks with vehicle (n = 9), oxaliplatin (n = 7), oxaliplatin plus MAG (n = 9) or MAG alone (n = 8). Each group was compared to animals injected with vehicle, and differences were considered significant when $P < 0.05$ ($^{*}p < 0.05$; $^{**}p < 0.01$; $^{***}p < 0.001$). The oxaliplatin-induced effects are highlighted in grey. Note that, when compared to vehicle-treated mice, all the variables modified in oxaliplatin-injected mice remain constant when oxaliplatin plus MAG or MAG alone was administered. (A) Strength-duration relationship. (B) Threshold electrotonus in response to constant depolarizing (up) and hyperpolarizing (down) long-duration currents applied at sub-threshold intensity (±20%, ±40%, ±70%). (C) Current-threshold relationship. (D) Recovery cycle.
